# Identifying the Drivers of Inter-Regional Patients’ Mobility: An Analysis on Hospital Beds Endowment

**DOI:** 10.3390/healthcare11142045

**Published:** 2023-07-17

**Authors:** Giovanni Guarducci, Gabriele Messina, Simona Carbone, Nicola Nante

**Affiliations:** 1 Post Graduate School of Public Health, University of Siena, 53100 Siena, Italy; gabriele.messina@unisi.it (G.M.); nicola.nante@unisi.it (N.N.); 2 Department of Molecular and Developmental Medicine, University of Siena, 53100 Siena, Italy; 3 General Directorate for Health Planning, Ministry of Health, 01144 Rome, Italy; s.carbone@sanita.it

**Keywords:** patients’ mobility, beds endowment, Gandy’s Nomogram, healthcare management, healthcare services, hospital rehabilitation, Italian regions

## Abstract

Background: In a Beveridgean decentralized healthcare system, like the Italian one, where regions are responsible for their own health planning and financing, the analysis of patients’ mobility appears very interesting as it has economic and social implications. The study aims to analyze both patients’ mobility for hospital rehabilitation and if the beds endowment is a driver for these flows; Methods: From 2011 to 2019, admissions data were collected from the Hospital Discharge Cards database of the Italian Ministry of Health, population data from the Italian National Institute of Statistics and data on beds endowment from the Italian Ministry of Health website. To evaluate patients’ mobility, we used Gandy’s Nomogram, while to assess if beds endowments are mobility drivers, we created two matrices, one with attraction indexes (AI) and one with escape indexes (EI). The beds endowment, for each Italian region, was correlated with AI and EI. Spearman’s test was carried out through STATA software; Results: Gandy’s Nomogram showed that only some northern regions had good hospital planning for rehabilitation. A statistically significant correlation between beds endowment and AI was found for four regions and with EI for eight regions; Conclusions: Only some northern regions appear able to satisfy the care needs of their residents, with a positive attractions minus escapes epidemiological balance. The beds endowment seems to be a driver of patients’ mobility, mainly for escapes. Certainly, the search for mobility drivers needs further investigation given the situation in Molise and Basilicata.

## 1. Introduction

In recent years, globalization and the resulting growth in the market for healthcare have promoted patients’ travel between countries to access healthcare services [1,2]. The healthcare services for which patients are willing to travel range from fertility treatments to dental care and various types of surgery [3]. Consequently, the analysis of cross-border patients’ mobility has lately become a hot topic for policy makers and researchers from different disciplines [4,5,6]. Even though the focus on international dynamics masks the fact that patient mobility often occurs within national borders, this is particularly evident in decentralized, tax-funded healthcare systems characterized by significant long stories of socio-economic disparities between regions, such as in Italy [4,7,8]. Then, the analysis of this phenomenon not only involves aspects such as the quality of health services (real or perceived) and equity of access but also has important economic implications [9,10,11]. In Italy, the National Health Service (NHS), established in 1978, is financed through general taxation at the central level, while planning and resource allocation takes place at the local level. Since the 1990s, the NHS has experienced a strong process of decentralization, ending in 2001 with a constitutional reform that established fiscal decentralization for the health sector. So, the NHS was restructured into several regional systems, each responsible for planning services and allocating resources and financing them [8,12,13]. These reforms have set up a quasi-market system in which the health services of each region are in potential competition with each other [9,14]. The process of decentralization has produced mixed results, as some regions have taken advantage of it to strengthen their systems, while others have been unable to develop an effective steering role [12]. In this context of competition, where the patient is free to choose, the study of these flows appears to be very interesting as their determinants could challenge universalism and equity of access at the national level [15,16]. Today, the factors influencing the choice of place of care are heterogeneous [17,18]. They include the socio-economic status [19], the patient’s clinical severity [20,21], the reputation of the hospital department and direct knowledge of the physician working there [22], the waiting times [23], and the distance and clinical quality of the hospital [24]. Other factors influencing patient–service interaction include technological equipment [25], the level of specialization and the number of doctors per patient [26,27]. Finally, recent studies have highlighted the importance of regulatory issues in driving patients’ mobility [10,14]. However, despite the flourishing literature, little is still known about the process that leads to choosing a specific place of care [28].

The study aims are (i) to evaluate the fulfilment of needs on site and patients’ mobility for hospital rehabilitation and (ii) to investigate if beds endowment drives patients’ mobility.

## 2. Materials and Methods

### 2.1. Admissions, Beds Endowment, Population Data and Catchment Area

Admissions data were collected from the Hospital Discharge Cards (HDCs) database of the Italian Ministry of Health from 2011 to 2019. We included the hospitalization of Italian patients for hospital rehabilitation for all Major Diagnostic Categories (MDCs). We excluded the hospitalization of patients residing in other states and the admissions of Italian patients to a foreign hospital. Hospital beds endowment data were collected from the Italian Ministry of Health website, which publishes a report with the total number of beds endowment divided by year, region and type of activity (acute, rehabilitation and long-term care) [29]. We included all beds endowment only for the hospital rehabilitation activity. Population data were extracted from “Health for All”, a database periodically released by the Italian National Institute of Statistics [30]. To indicate the number of beds per 1000 inhabitants for region Xi (with i = 1, …, 21) and for year Yi (i = 2011, …, 2019), the following formula was calculated:

NB × 1000 inhabitants Xi/Yi = TB × 1000/TP

where NB *×* 1000 inhabitants Xi/Yi = number of beds *×* 1000 inhabitants for region Xi and for year Yi; TB = total beds endowment in the area Xi and for the year Yi; TP = total population in the year Yi.

As catchment areas, we considered individually those of the 21 single Italian regional healthcare services (19 regional plus the Autonomous Province of Trento and the Autonomous Province of Bolzano). For each catchment area, the healthcare mobility flows were carried out through data on hospitalization of residents in their own region, attractions from other regions (A) and escapes to other regions (E).

### 2.2. Gandy’s Nomogram

Hospitalization of R, A and E data were processed through Gandy’s Nomogram, which makes it possible to provide a brief representation of the access to the hospital facilities allocated in each catchment area. In fact, it is useful for comparing many geographical areas in a single representation and allows trends over time to be analyzed [9,13,31,32,33]. Gandy’s Nomogram is a squared area placed in a Cartesian plan with the side of 100; one can be divided into four squares by two lines parallel to the two axes, which start at X = 0, Y = 50 and X = 50; Y = 0. A diagonal divides the plan in an upper area, in which the Y value is higher than the X one; in this part of the Cartesian plan, there are more attractions than escapes. The lower area shows the opposite situation. The diagonal starts from the point named “O” with coordinates X = 0 and Y = 0 and ends at the point named “W” with coordinates X = 100 and Y = 100. At the point O, the attractions have a maximum value, while at the point W they are null. When the point falls on the diagonal, the values of attractions and escapes are equal.

The value on the *x*-axis (X) indicates residents (R) out of residents (R) plus attractions (A) as in the following formula:

X = R/(R + A) × 100

The value on the *y*-axis (Y) indicate residents (R) out of residents (R) plus escapes (E) as in the following formula:

Y = R/(R + E) × 100

The four quadrants obtained in the Cartesian plan show the different capacity of a catchment area to satisfy the needs of their residents and its epidemiological balance, attractions minus escapes:

₋ The upper-left quadrant describes the catchment areas “marked oriented”, in which are admitted more patients attracted than residents and escapes are less than attractions and residents admission (E < R < A). At the point X = 0 and Y = 100 is shown the paradoxical condition in which are admitted only attracted patients and there are no escapes.
₋ The upper-right quadrant describes the catchment areas which can meet the care needs of their residents on site. In the part above the bisector, named “hemi-quadrant of quality), attractions are more than escapes and the latter less than residents’ admissions (E < A < R). In the part under the bisector, escapes are more than attractions and the latter are less than residents’ admissions.
₋ The lower-left quadrant describes the catchment areas in which the residents’ admissions are less than both escapes and attractions. In the part above the bisector, escapes are less than attractions (R < E < A), while in the part under the bisector is shown the opposite situation (A < R < E).
₋ The lower-right quadrant describes the catchment areas in which the residents’ admissions are less than escapes but greater than attractions (A < R < E).

### 2.3. Attraction and Escape Indexes

To measure the incidence of attractions and escapes in a region, we used the Attraction Index (AI) and the Escape Index (EI) [34]. The AI indicates the percentage of attractions (A) out of the total admissions in the region (A + R) in the following formula:

AI = A/(R + A) × 100

The EI indicates the percentage of escapes (E) out of the total discharges of residents wherever hospitalized (R + E) in the following formula:

EI = E/(R + E) × 100.

These two indexes allow us to quantify both a region’s capacity to attract patients (AI) and the propensity of its citizens to leave (EI).

### 2.4. Graphical Representation of AI, EI and NB × 1000 Inhabitants

From these two indexes (AI and EI) and the NB × 1000 inhabitants, we created two matrix models, one for attractions and one for escapes. For both models, we designed a cartesian plan. In the *x*-axis was always the NB × 1000 inhabitants. In the *y*-axis, the first model was the AI, while the second was EI. In both cartesian planes, the two axes were intersected at the national mean of NB *×* 1000 inhabitants and the corresponding national mean of the index (AI or EI) [1].

### 2.5. Vectorial and Statistical Analysis

For every region, both for Gandy’s Nomogram and for the two graphical representations mentioned above, the points born from the coordinates of the X and Y axis were linked to obtain a vector. If there was a monophasic trend over the years (same direction of the points), 2011 was linked to 2019. If there was a biphasic trend over the years (change of law), 2011 was linked to the point of changing direction, which was linked to 2019 [13,31,35]. The STATA software SE/14.0 (StataCorp LLC, College Station, TX, USA) was used for data management and for statistical analysis. Trends were studied with Cuzick’s test, and the difference in the ORD/DH ratio between residents and mobility admissions was studied with the Mann–Whitney U-test. Finally, the correlations between AI, EI and NB × 1000 inhabitants were studied through Sperman’s rank correlation test. The level of significance was set up at the level of 95% (*p* < 0.05).

## 3. Results

Table 1 shows the admissions, in ordinary regime (ORD) and day hospital (DH), for hospital rehabilitation divided into residents and mobility from 2011 to 2019. In the studied period, the total number of admissions decreased (*p* < 0.05), even if the hospitalization in mobility increased (*p* < 0.05). The hospitalizations in ORD increased (*p* < 0.05) for mobility admissions, while for residents, after the initial increase, they decreased to lower values than at the start. The admissions in DH decreased (*p* < 0.05) both for residents and for mobility. Thus, the ORD/DH ratio increased (*p* < 0.05). Moreover, ORD/DH ratios varied if admissions were in mobility or for residents (*p* < 0.05).

**Figure 1:**
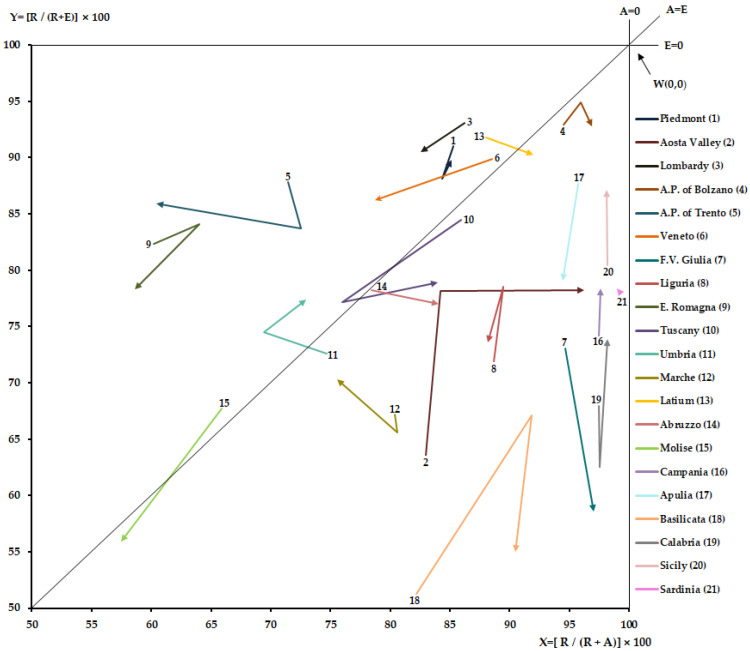
Upper-right quadrant of Gandy’s Nomogram, Italian regions 2011–2019.

Figure 1 shows the upper-right quadrant of Gandy’s Nomogram for each Italian region for hospital rehabilitation from 2011–2019. Only Piedmont, Lombardy, A.P. of Trento, Veneto, and E. Romagna were in the hemi-quadrant of quality for all studied periods. Molise and Latium lost their good position in the last years of the studied period, while Umbria improved it. Tuscany had bypassed the bisector, only to return to the starting hemi-quadrant in 2018. The attractions increased significantly for Lombardy, A.P. of Trento, Veneto, and Molise, while they decreased significantly for Aosta Valley, A.P. of Bolzano, F.V. Giulia, Abruzzo, and Basilicata (*p* < 0.05). Escapes decreased significantly for Aosta Valley, Campania, Calabria, and Sicily, while they increased substantially for Veneto, F.V. Giulia, E. Romagna, Tuscany, Lazio, Molise, and Apulia (*p* < 0.05).

Table 2 shows the number of beds (NB) × 1000 inhabitants for hospital rehabilitation, for every Italian region for the years from 2011 to 2019. At the national level, the average value of NB × 1000 inhabitants was 0,49 with a higher value in 2019 and lower in 2012, 2013 and 2015. It was higher for Piedmont, Aosta Valley (except in 2011), Lombardy, A.P. of Trento, Latium, and Molise. In the studied period, the NB × 1000 inhabitants increased significantly (*p* < 0.05) for A.P. of Bolzano, Tuscany, Umbria, Marche, and Sicily, while it decreased significantly (*p* < 0.05) for E. Romagna and Latium.

**Figure 2:**
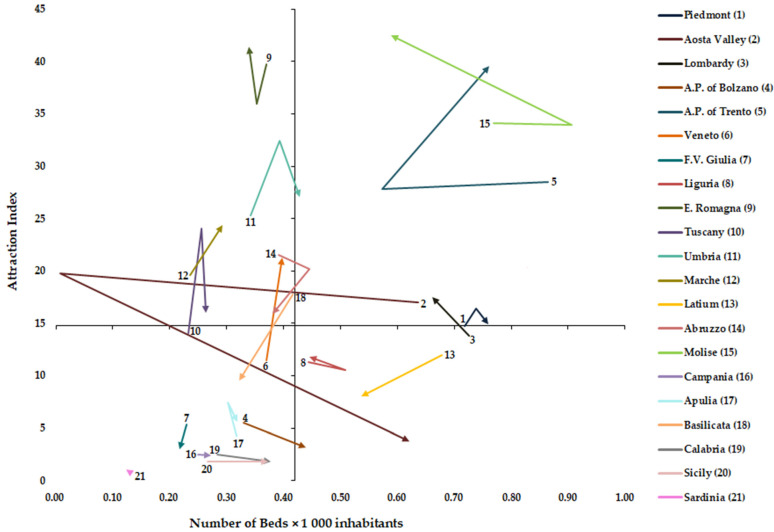
Graphical representation of Number of Beds × 1000 inhabitants and Attraction Index.

Figure 2 shows the matrix between NB × 1000 inhabitants and Attraction Index (AI) from 2011 to 2019. The AI increased significantly (*p* < 0.05) for Lombardy, A.P. of Trento, Veneto, and Apulia, while it decreased for Aosta Valley, A.P. of Bolzano, F.V. Giulio, Abruzzo, and Basilica (*p* < 0.05). A statistically significant correlation between NB × 1000 inhabitants and AI was for Veneto (Spearman’s rho = 0.7000), Latium (Spearman’s rho = 0.7333), Molise (Spearman’s rho = −0.8667) and Basilicata (Spearman’s rho = 0.8500).

**Figure 3:**
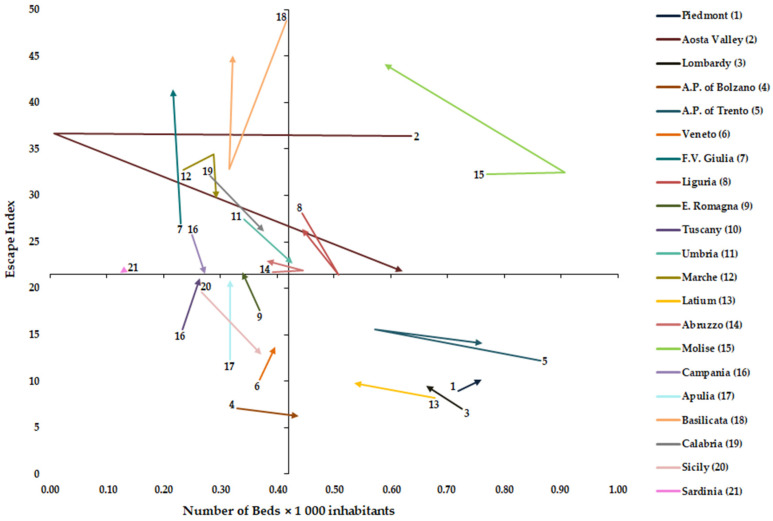
Graphical representation of Number of Beds × 1000 inhabitants and Escape Index.

Figure 3 shows the matrix between NB × 1000 inhabitants and Escape Index (EI) from 2011 to 2019. The EI decreased significantly (*p* < 0.05) for Aosta Valley, Campania, Sicily, and Calabria, while it increased for Lombardy, Veneto, F.V. Giulia, E. Romagna, Tuscany, Latium, Molise, and Apulia (*p* < 0.05). A statistically significant correlation between NB × 1000 inhabitants and EI was found for A.P. of Bolzano (Spearman’s rho = −0.667), E. Romagna (Spearman’s rho = −0.7000), Umbria (Spearman’s rho = −0.6667), Latium (Spearman’s rho = −0.8333), Molise (Spearman’s rho = −0.7333), Basilicata (Spearman’s rho= 0.7667), Calabria (Spearman’s rho = −0.7776) and Sicily (Spearman’s rho = −0.9500).

## 4. Discussion

Cross-border patients’ mobility is becoming a predominant phenomenon for the reallocation of healthcare resources between countries, regions and provinces [36]. In Italy, approximately one in ten hospital admissions are due to patients’ mobility, and this phenomenon indirectly indicates how the Italian NHS’s mission is being pursued: to meet the care needs of its residents on site [9,37]. Some flows may be considered “physiological” because they are due either to shifts of patients between bordering areas or to the size of the catchment areas with high-specialty hospitals: the latter may pose a threat to equity in that patients from lower socio-economic groups may find it difficult to bear the costs of moving [19]. Other flows must be considered ‘pathological’ since they are related to both qualitative and quantitative (real/perceived) inadequacy of the healthcare offer on site: this might cause discomfort to the citizen who must turn to healthcare facilities outside his area to obtain better conditions in terms of quality and accessibility of care [9,38]. Low hospital quality may persist in the long run because the local population may find good hospital quality elsewhere. Therefore, it is essential for the national government to implement some policies that can improve the quality of hospitals in regions with low hospital quality. In fact, an NHS with asymmetrical quality of healthcare in different regions could generate social problems for the local population [39]. However, it is not easy to imagine a healthcare system that provides all types of services, especially those of high complexity, in every territorial context. This study aims to assess how the various regional health services respond to the demand for rehabilitation hospital services and the inter-regional patients’ mobility for these flows. In addition, we analyzed whether there is a correlation between the rehabilitation hospital beds endowment and the mobility flows studied.

During the period studied, the demand for total hospital rehabilitation decreased, while the one for orthopedic intensive rehabilitation increased [31] due to a significant reduction in DH admissions. The increase in rehabilitation admissions in ORD does not justify such a high decrease in DH. This logically caused an increase in the ORD/DH ratio of both resident and mobility admissions; for the latter, it was more pronounced. The reasons could be due to a more strategic allocation of resources and a consequently more efficient reorganization of outpatient rehabilitation activity [40], thus leading to a shift of demand to the outpatient level. Indeed, the outpatient system plays a central role in meeting the population’s needs at a more local level [41]. At the same time, mobility admissions increased (+2% approximately), showing higher values than “acute” hospital admissions [9].

Although all regions appear to be able to meet the rehabilitation needs of their residents on site, Gandy’s Nomogram shows a heterogeneous pattern with only a few areas in northern Italy with a positive epidemiological balance (attractions minus escapes) for all studied years. Umbria, in contrast to Latium, in addition to having implemented attractions for a few years, seems to have started a process of escape recovery. Other regions, such as Tuscany and Molise, had implemented attractions, but this was not enough to keep a positive epidemiological balance due to the increase in escapes. The islands and some southern regions show low attractiveness, but they, too, have started on a path of decreasing escapes.

In the analysis of mobility drivers, it is important to differentiate the factors that can be controlled by policy makers (quality, regulation issues, and availability of resources) and those minimally influenced by health policies (macroeconomic, social, and individual factors) [1]. Higher values of NB × 1000 inhabitants were observed for four regions in the north (Piedmont, Aosta Valley, Lombardy and A.P. of Trento), one in the center (Latium) and one in the south (Molise). Aosta Valley, Lombardy and Molise show for hospital rehabilitation above-average values of beds as well as for acute one [9]. Certainly, these regions have a strong tendency to keep their beds endowment high. Over the period studied, a significant increase in rehabilitation beds has been observed for five regions (A.P. of Bolzano, Tuscany, Umbria, Marche, and Sicily), although this does not indicate a higher quality of care [1] but could indicate a better response to the demand for care from their citizens. In our work, for some areas, in contrast to the results of another study [42], the beds endowment is correlated with the choice of the place of care. Specifically, we found a positive correlation between AI and beds endowment for three regions (Veneto, Latium, and Basilicata), while Molise shows a negative correlation. With EI, a negative correlation, as logical, was found for seven regions (A.P. of Bolzano, E. Romagna, Umbria, Latium, Molise, Calabria, and Sicily), while a positive correlation was found for Basilicata. This heterogeneity may be due to the different characteristics of the various regional systems in which the role of competition appears very different. In fact, in a more centralized system such as that of E. Romagna, all hospitals are perceived as homogeneous providers, and therefore patients are less aware of differences in clinical quality. Whereas in other more decentralized contexts, where competition between providers is more aggressive, patients are more aware that quality is different between them [28]. The situation found in Basilicata and Molise, where the latter region also has bed values above the national average, might make us reflect on the fact that in smaller regions where the whole territory is a ‘border’, it is necessary to investigate other factors influencing patient mobility. These are probably not to be found in the structural endowment of their hospitals, but, for example, should be found in the waiting times or in the availability of services in a specific area of the region where a particular health service is not provided [18].

Our study contributes to the literature by highlighting the role of beds endowment in the dynamics of inter-regional mobility, with a view to pointing out possible health policy initiatives aimed at improving the quality and accessibility of healthcare services in hospital rehabilitation.

### Limitations

This study has some limitations: (i) the data analyzed predates the pandemic period; we chose not to analyze 2020 (latest available data) because COVID-19 changed healthcare planning and organization, and therefore there may have been a new organizational setting during that period in terms of beds utilization; (ii) we analyzed hospital rehabilitation in its totality without breaking it down by pathologies; (iii) we did not quantify proximity mobility (movements from areas near regional borders), but we considered the flows in total.

## 5. Conclusions

Inter-regional patients’ mobility for hospital rehabilitation is a phenomenon that has increased in recent years, so it is crucial to understand its drivers. The results show that some northern regions seem to meet the needs of their residents better, possibly due to their higher beds endowment, and perform better in attracting patients. However, outflows for some areas seem to have decreased on the islands and in some southern regions, which shows low attractiveness. In the long run, this phenomenon, constantly growing, could generate even more regional disparities and risk causing discrimination between different citizens in different regions, especially those with lower socio-economic status.

For some regions, the beds endowment seems to drive patients’ mobility. Notably, we observed that a decrease in the endowment is correlated with an increase in escapes to extra-regional hospitals in one-third of the Italian regions. For these regions, the inverse association between these two variables could seriously affect the accessibility of hospital rehabilitation services, also considering that once a negative trend is started it is not easy to stop or reverse. Indeed, the drivers of patients’ mobility need further investigation, especially considering the situation in Molise and Basilicata.

We believe the tools used to analyze the correlation between mobility indicators and beds endowment can provide valuable insight for policy makers. Furthermore, they could be applied in relating other structural factors, such as the number of doctors per patient, both to provide easy-to-read indicators to health policy makers and to identify possible other drivers of patients’ mobility.

## Figures and Tables

**Table 1:** Patients’ admissions to Italian hospitals for rehabilitation, 2011–2019.

Year	Residents			Mobility			Total			Total (ORD + DH) *
	ORD	DH *	ORD/DH *	ORD *	DH *	ORD/DH *	ORD	DH *	ORD/DH *	
2011	263,884	47,550	5.55	44,067	5733	7.69	307,951	53,283	5.78	361,234
2012	266,029	39,783	6.69	46,069	5328	8.65	312,098	45,111	6.92	357,209
2013	269,373	36,419	7.40	48,132	4452	10.81	317,505	40,871	7.77	358,376
2014	265,473	31,692	8.38	48,462	3173	15.27	313,935	34,865	9.00	348,800
2015	267,010	30,812	8.67	50,466	3236	15.60	317,476	34,048	9.32	351,524
2016	265,753	28,905	9.19	51,796	2949	17.56	317,549	31,854	9.97	349,403
2017	263,474	27,716	9.51	51,579	3001	17.19	315,053	30,717	10.26	345,770
2018	260,856	25,477	10.24	50,596	2974	17.01	311,452	28,451	10.95	339,903
2019	261,481	25,524	10.24	49,386	3092	15.97	310,867	28,616	10.86	339,483

* statistically significant trend (*p* < 0.05).

**Table 2:** NB × 1000 inhabitants for hospital rehabilitation, Italian region 2011–2019.

Region	2011	2012	2013	2014	2015	2016	2017	2018	2019	Mean 2011–2019
Piedmont	0.72	0.75	0.75	0.74	0.74	0.74	0.74	0.75	0.76	0.74
Aosta Valley	0.64	0.01	0.51	0.61	0.59	0.59	0.59	0.62	0.62	0.53
Lombardy	0.73	0.67	0.65	0.65	0.66	0.67	0.66	0.66	0.66	0.67
A.P. of Bolzano *	0.33	0.33	0.34	0.37	0.37	0.43	0.42	0.44	0.35	0.37
A.P. of Trento	0.86	0.57	0.80	0.75	0.69	0.69	0.71	0.70	0.76	0.73
Veneto	0.37	0.37	0.37	0.37	0.38	0.40	0.40	0.40	0.40	0.38
F.V. Giulia	0.23	0.22	0.24	0.24	0.23	0.24	0.23	0.23	0.22	0.23
Liguria	0.44	0.41	0.48	0.51	0.43	0.46	0.45	0.45	0.44	0.45
E. Romagna *	0.37	0.37	0.36	0.35	0.35	0.35	0.34	0.34	0.34	0.35
Tuscany *	0.23	0.24	0.24	0.25	0.26	0.25	0.24	0.26	0.26	0.25
Umbria *	0.34	0.35	0.39	0.39	0.39	0.40	0.42	0.43	0.43	0.39
Marche *	0.24	0.24	0.25	0.25	0.26	0.29	0.29	0.29	0.29	0.27
Latium *	0.68	0.59	0.56	0.56	0.55	0.54	0.54	0.53	0.54	0.56
Abruzzo	0.39	0.39	0.39	0.38	0.38	0.45	0.38	0.39	0.38	0.39
Molise	0.77	0.75	0.91	0.78	0.77	0.77	0.65	0.65	0.59	0.74
Campania	0.25	0.24	0.25	0.25	0.24	0.25	0.25	0.25	0.27	0.25
Apulia	0.32	0.30	0.31	0.31	0.30	0.30	0.30	0.30	0.32	0.31
Basilicata	0.42	0.33	0.34	0.34	0.34	0.32	0.32	0.32	0.32	0.34
Calabria	0.28	0.26	0.32	0.41	0.37	0.36	0.40	0.41	0.38	0.36
Sicily *	0.27	0.29	0.31	0.32	0.32	0.32	0.32	0.35	0.37	0.32
Sardinia	0.13	0.13	0.10	0.13	0.13	0.13	0.13	0.13	0.12	0.13
Italy	0.49	0.48	0.48	0.49	0.46	0.49	0.49	0.49	0.50	0.49

* statistically significant trend (*p* < 0.05).

## Data Availability

Data obtained from HDCs database—General Directorate for Health Planning of Italian Ministry of Health upon specific request.
